# An Anthelmintic Drug, Pyrvinium Pamoate, Thwarts Fibrosis and Ameliorates Myocardial Contractile Dysfunction in a Mouse Model of Myocardial Infarction

**DOI:** 10.1371/journal.pone.0079374

**Published:** 2013-11-04

**Authors:** Motoaki Murakoshi, Kyohei Saiki, Kyoji Urayama, Thomas N. Sato

**Affiliations:** 1 Graduate School of Biological Sciences, Nara Institute of Science and Technology, Nara, Japan; 2 Department of Biomedical Engineering, Cornell University, Ithaca, New York, United States of America; 3 Centenary Institute, Sydney, Australia; University of Otago, New Zealand

## Abstract

Metabolic adaptation to limited supplies of oxygen and nutrients plays a pivotal role in health and disease. Heart attack results from insufficient delivery of oxygen and nutrients to the heart, where cardiomyocytes die and cardiac fibroblasts proliferate – the latter causing scar formation, which impedes regeneration and impairs contractility of the heart. We postulated that cardiac fibroblasts survive metabolic stress by adapting their intracellular metabolism to low oxygen and nutrients, and impeding this metabolic adaptation would thwart their survival and facilitate the repair of scarred heart. Herein, we show that an anthelmintic drug, Pyrvinium pamoate, which has been previously shown to compromise cancer cell survival under glucose starvation condition, also disables cardiac fibroblast survival specifically under glucose deficient condition. Furthermore, Pyrvinium pamoate reduces scar formation and improves cardiac contractility in a mouse model of myocardial infarction. As Pyrvinium pamoate is an FDA-approved drug, our results suggest a therapeutic use of this or other related drugs to repair scarred heart and possibly other organs.

## Introduction

Ischemic heart disease accounts for ~13 % of deaths worldwide and is the leading cause of death for both men and women in all developed countries. Myocardial infarction, commonly known as a heart attack, is primarily caused by the occlusion of blood supply to a part of the heart, causing the cardiomyocytes to die. Cardiomyocyte death is followed by fibrosis (i.e., scar formation) resulting from active proliferation and migration of cardiac fibroblasts and excessive deposition of extracellular collagen fibers [1,2]. Cardiac fibrosis has adverse effects on cardiac function, and furthermore, interferes with regeneration of cardiomyocytes and cardiac vascularization, the essential processes for restoring function of scarred heart [1,2]. Thus, an effective therapeutic intervention of cardiac fibrosis is very much in need for full regeneration of the heart injured by heart attack and other cardiac diseases. However, this therapeutic goal remains unmet despite several decades of extensive studies. 

Conventional therapeutic approaches towards interfering with cardiac fibrosis have been to inhibit proliferation and/or migration of cardiac fibroblasts [1,2]. These include targeted inhibition of activities of fibrogenic cytokines/proteins such as transforming growth factor beta (TGF-β), connective tissue growth factor (CTGF/CCN2), platelet-derived growth factor (PDGF) and endothelin-1. Furthermore, unusually elevated intracardial level of angiotensin II (Ang II) was found in overloaded hearts with fibrosis and that drugs inhibiting angiotensin signaling pathway has been used to reduce cardiac fibrosis [3,4]. However, none have lead to desirable therapeutic consequences. Thus, as a completely new class of therapeutic targets, we considered altered intracellular metabolism in cardiac fibroblasts under ischemia. We hypothesized that, in ischemic heart diseases, cardiac fibroblasts survive and actively proliferate in a metabolically challenging microenvironment with limited amounts of oxygen and nutrients. Based on this hypothesis, we postulate that impeding such metabolic adaptation in cardiac fibroblasts serves as a novel and effective therapeutic target to attenuate cardiac fibrosis in ischemic heart diseases including myocardial infarction. If successful, this would become an important step towards curing this life threating and most prevalent human disease.

An anthelmintic drug, pyrvinium pamoate (PP), is an inhibitor of NADH-fumarate reductase (NADH-FR) activity in the anaerobic respiratory chain in mitochondria of parasitic worm [5]. In cancer cells, it has been recently reported that PP also inhibits canonical Wnt, unfolded protein response (UPR), androgen receptor and autophagy signals [6-10]. It has been also shown that PP can compromise the survival of cancer cells under glucose-starvation condition [11-13]. These previously studies suggested to us that PP could potentially starve cardiac fibroblasts to death in glucose-deficient microenvironment and thwart cardiac fibrosis.

In this study, we show that PP can in fact disables the survival of cardiac fibroblasts specifically under glucose-deficient media in vitro and also thwarts fibrosis and ameliorate myocardial contractile dysfunction in a mouse model of myocardial infarction.

## Results and Discussion

**Figure 1:**
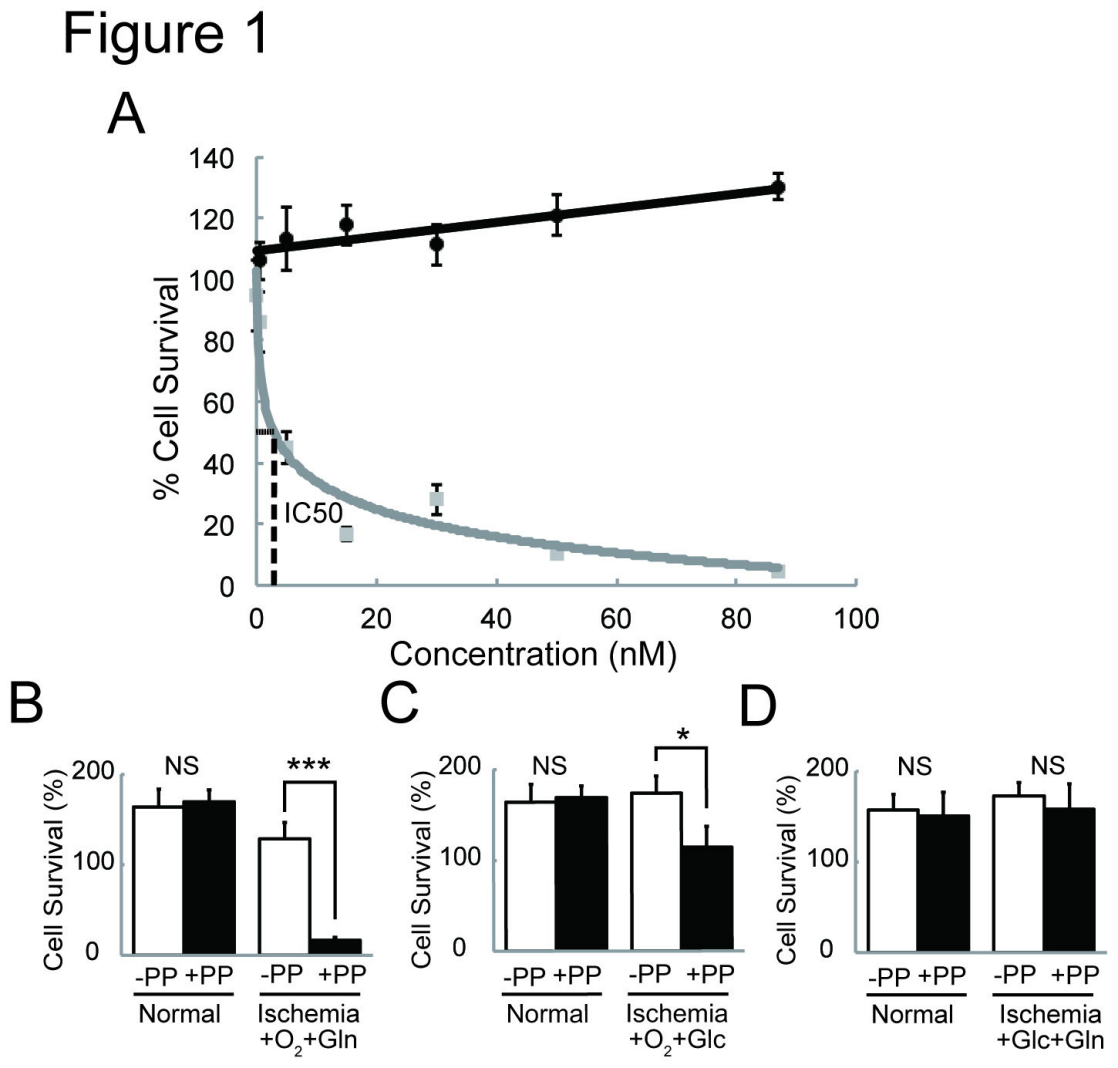
Cytotoxic effect of pyrivinium pamoate (PP) on cardiac fibroblasts. **A**. Cytotoxic effect of pyrvinium pamoate (PP). Cytotoxic effects of pyrvinium pamoate (PP), on cardiac fibroblasts under normal (Black line) and ischemic (Grey line) conditions are shown. IC50 for PP on cardiac fibroblasts under O_2_^low^/Glc^low^/Gln^low^ culture condition (Ischemia) is 9.5 nM. Cells (n=3) were treated with each drug at each concentration for 72 hr under either normal (black line) or O_2_^low^/Glc^low^/Gln^low^ (ischemia) (grey line) conditions. Data are shown as % of cell numbers of each culture condition without drug treatment. **B**. The effect of oxygen (+O_2_) and glutamine (+Gln) supplementation on the O_2_^low^/Glc^low^/Gln^low^ (ischemia)-specific cytotoxic effect of PP. **C**. The effect of oxygen (+O_2_) and glucose (+Glc) supplementation on the O_2_^low^/Glc^low^/Gln^low^(ischemia)-specific cytotoxic effect of PP. **D**. The effect of glucose (+Glc) and glutamine (+Gln) supplementation on the O_2_^low^/Glc^low^/Gln^low^(ischemia)-specific cytotoxic effect of PP. The results from the cells in each culture condition for 24 hr with and without PP (87 nM) are shown by empty and solid-filled boxes, respectively. The viable cell number at 0 hr under each culture condition is indicated as 100 %. Data: average±s.d. ^★^p<0.05, ^★★★^p<0.001. NS: not significant. n=3.

First, we tested cytotoxicity of PP on cardiac fibroblasts cultured under the limited oxygen, glucose and glutamine (3 % oxygen, 300 μM glucose, <100 μM glutamine) (referred to as O_2_^low^/Glc^low^/Gln^low^ or ischemia) and normal (20 - 21 % oxygen, 25 mM glucose, 4 mM glutamine) (referred to as “normal”) conditions for 72 hr (Figure 1A). PP was found to impose specific toxicity on cardiac fibroblasts in ischemia (IC50=9.5 nM) (Figure 1A). Furthermore, we found that the normal level of O_2_ failed to rescue the survival of cardiac fibroblasts treated with PP (Figure 1B). The survival of cardiac fibroblasts treated with PP in ischemia could not be rescued by normal levels of O_2_ (20 - 21%) and glutamine (4 mM) (Figure 1B). In contrast, their survival was rescued partially or completely by supplementing O_2_ (20 - 21%)/glucose (25 mM) (Figure 1C) or glucose (25 mM)/glutamine (4 mM) (Figure 1D), respectively. These results demonstrate that the cytotoxic effect of PP on cardiac fibroblasts specifically under glucose- and glutamine-deficient condition (Glc^low^/Gln^low^). Such PP effect on cardiac fibroblasts is similar to that on cancer cells [11].

**Figure 2:**
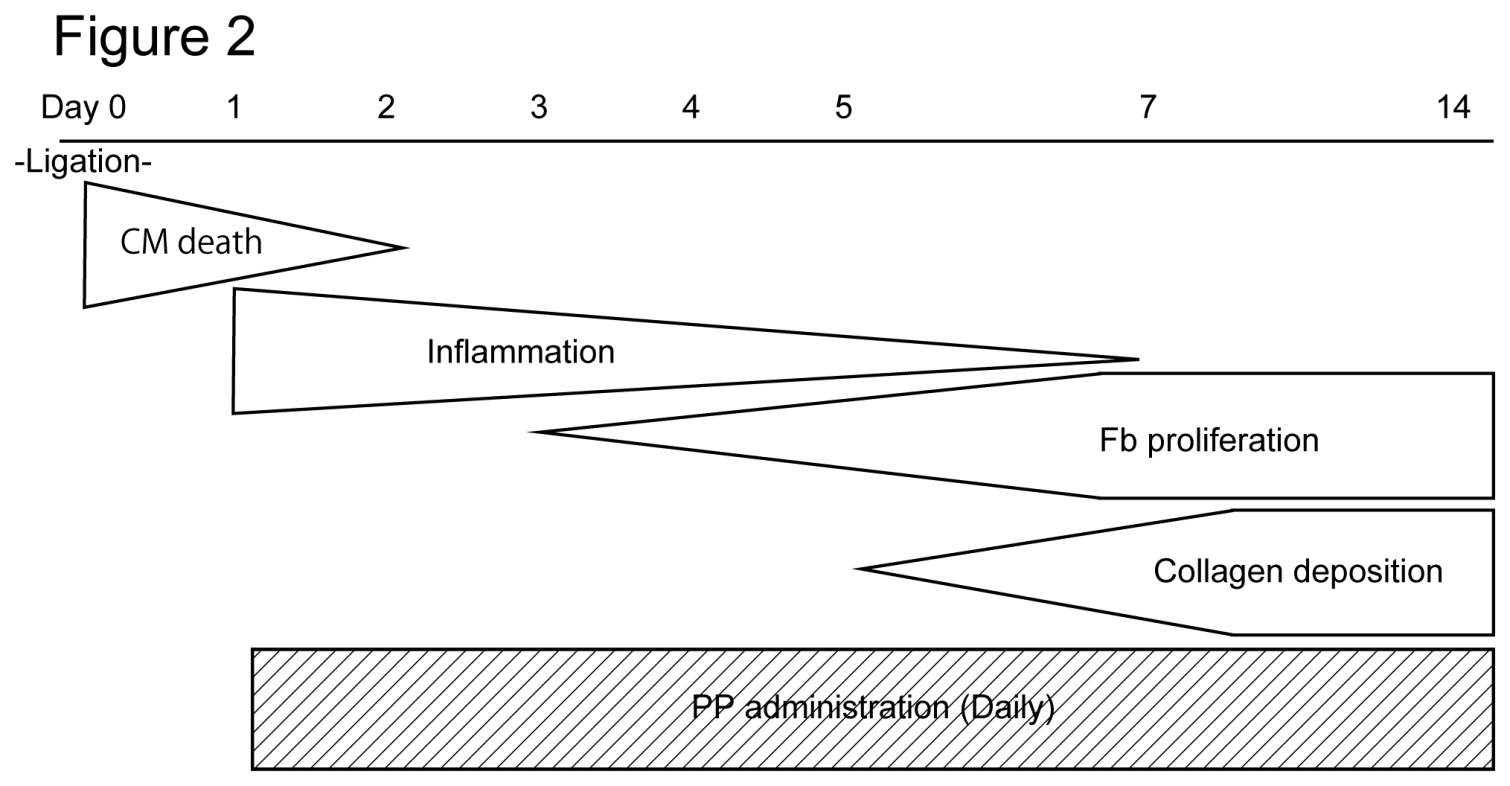
Schematic diagram of the pathological processes of the infarcted heart and the PP administration protocol. Within 24 hr following the ligation (or occlusion) of left coronary artery, most of cardiomyocytes (CM) die, which is followed by inflammation and fibrosis. Fibrosis is caused by proliferation and migration of cardiac fibroblasts (Fb) beginning at approximately day 3 post ligation (occlusion). These cardiac fibroblasts secrete collagen that forms collagen fibers and become deposited in the tissue and forms scar. The collagen deposition peaks at day 7 - 14 post-ligation (occlusion). PP was administered daily to mice after 1 day following the ligation of left coronary artery (i.e. after most of cardiomyocytes die).

**Figure 3:**
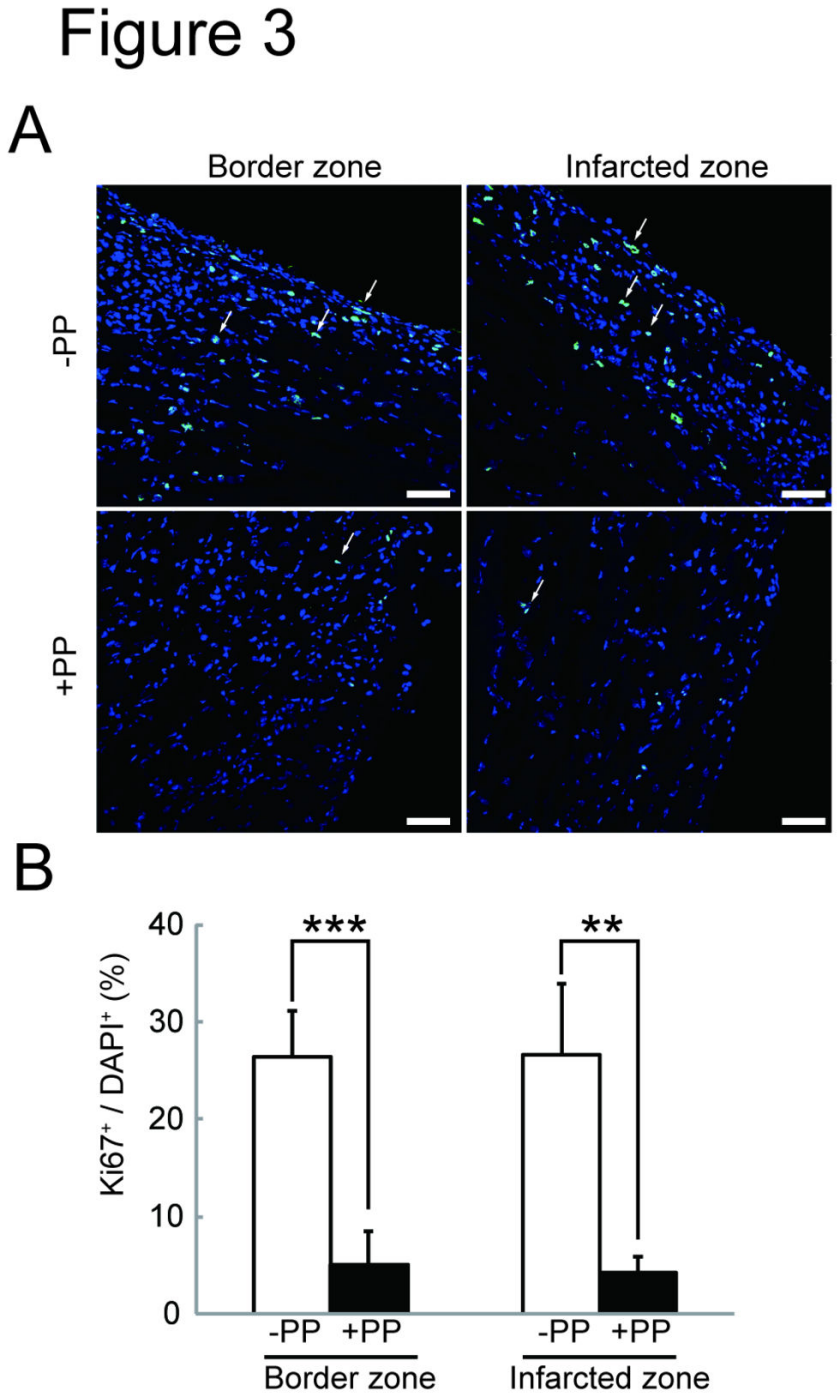
Decreased cell proliferation in the PP-treated infarcted heart in a mouse model of myocardial infarction. **A**. An effect of PP on cell proliferation in the infarcted heart. Ki67^+^ (green) and DAPI^+^ (dark blue) cells are identified by immunofluorescence. The proliferating Ki67^+^DAPI^+^ (light blue) cells in both border and infarcted zones of vehicle (DMSO) (-PP) or PP (+PP) treated mice are indicated (arrows). Scale bars: 50　μm. **B**. Quantitation of Ki67^+^ proliferating cells. The number of Ki67^+^ proliferating cells was counted from sections from mice (-PP: n=3; +PP: n=4) counted and shown as % (Ki67^+^ / DAPI^+^).

**Figure 4:**
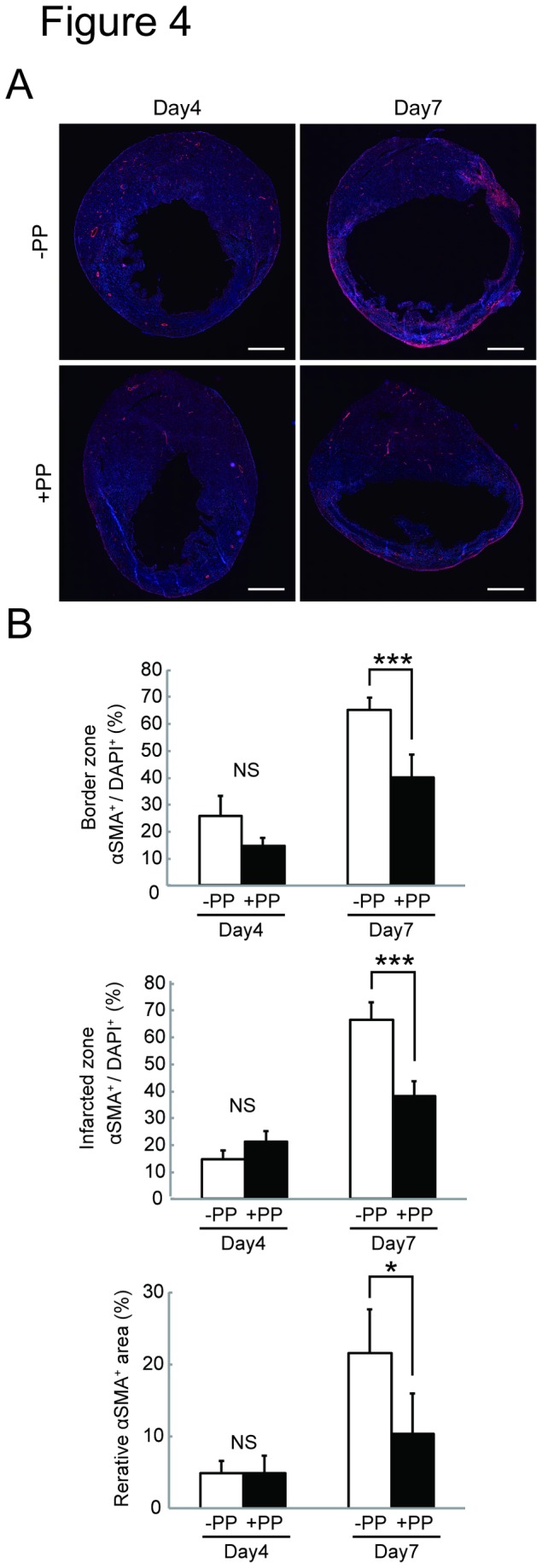
Decreased number of αSMA^+^ myofibroblasts in the PP-treated infarcted heart in a mouse model of myocardial infarction. **A**. An effect of PP on αSMA^+^ myofibroblasts in the infarcted heart. αSMA^+^ myofibroblasts (Red) and DAPI^+^ cells (dark blue) in the heart at day 4 and day 7 after the coronary artery ligation. The representative images from the mouse heart treated with vehicle (DMSO) (-PP) or PP (+PP) are shown. Scale bars: 1 mm. **B**. Quantitation of αSMA^+^ myofibroblasts. The number of αSMA^+^ myofibroblasts was counted from sections from vehicle treated (-PP) (n=3 mice for day 4, n=5 mice for day 7) and PP treated (+PP) (n=4 mice for day 4 and day 7) mice was counted in both border and infarcted zones and shown as % (αSMA^+^ / DAPI^+^). The αSMA^+^ area was also measured and shown as % (αSMA^+^ area / whole section area).

**Figure 5:**
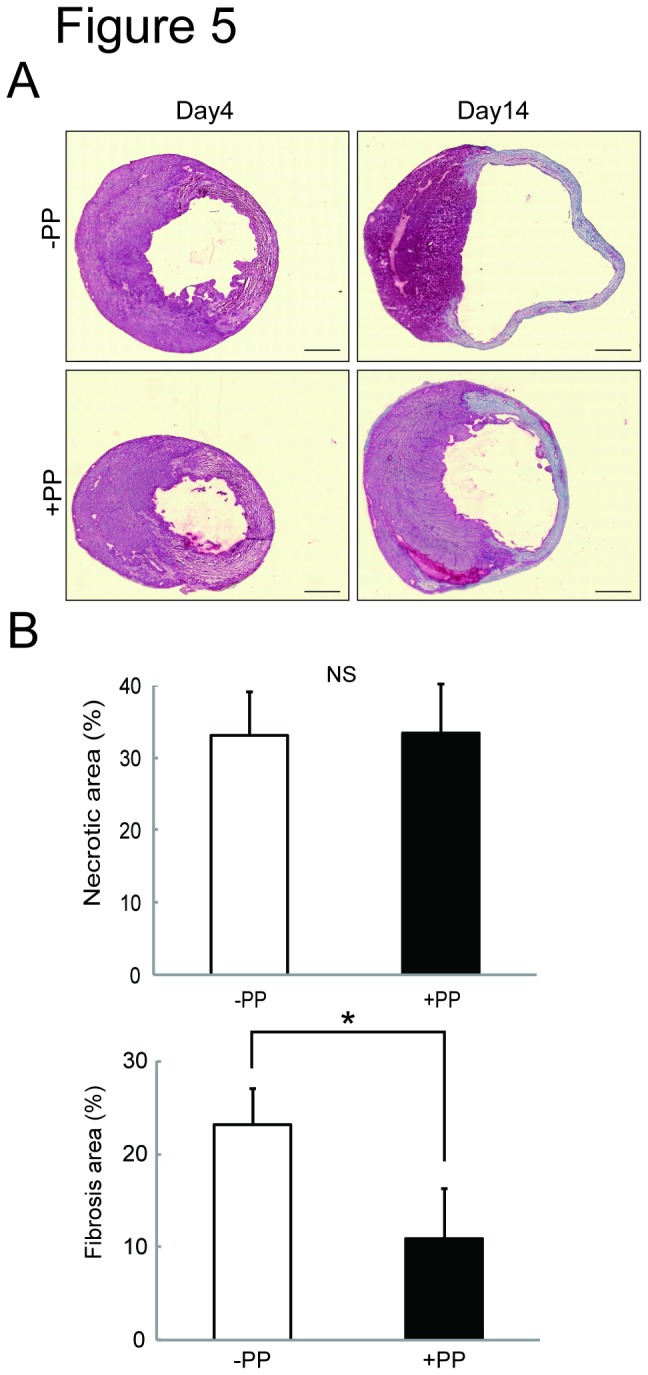
Reduced fibrosis in the PP-treated infarcted heart in a mouse model of myocardial infarction. **A**. Masson’s trichorme stained heart sections at day 4 and day 14 post-ligation. Scale bars: 1　mm. **B**. Quantitation of infarct size and fibrosis area. The infarct size was measured by quantitating the necrotic area of the heart sections prepared from the vehicle treated (-PP) (n=3 mice) and PP treated (+PP) (n=4 mice) mice at day 4 post-ligation, and shown as % (necrotic area / whole heart area) (left). The fibrosis size was measured by Masson’s trichrome staining method using the heart sections prepared from the vehicle treated (-PP) (n=3 mice) and PP treated (+PP) (n=4 mice) mice at day 14 post-ligation, and shown as % (fibrosis area stained blue / whole heart area).

**Figure 6:**
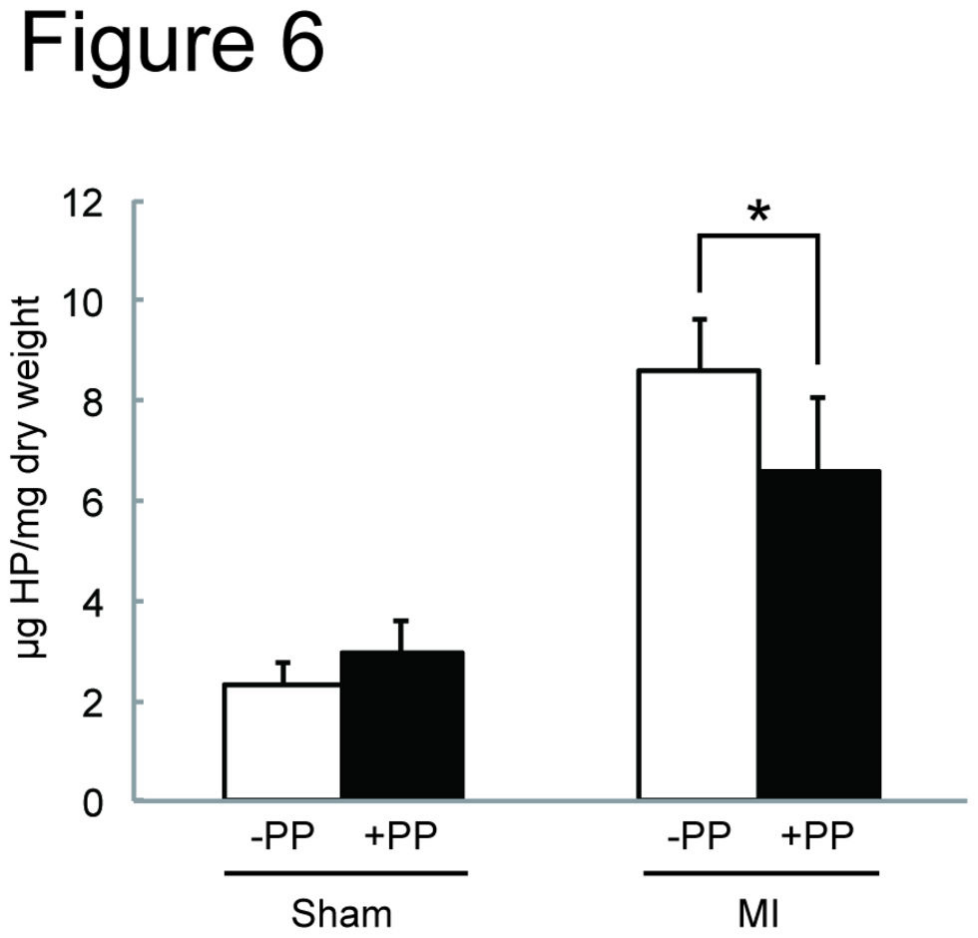
Decreased collagen deposition in the PP-treated infarcted heart in a mouse model of myocardial infarction. Quantitation of hydroxyproline (HP) contents in the left ventricle tissue. The left ventricle of the mice treated with vehicle (-PP) (n=4 mice for Sham, n=9 mice for MI) or with PP (+PP) (n=3 for Sham, n=10 for MI) were harvested from the mice at day 14 post sham-operation (Sham) or ligation (MI) and the hydroxyproline (HP) amounts were measure and shown as HP (μg)/mg dry tissue weight.

**Figure 7:**
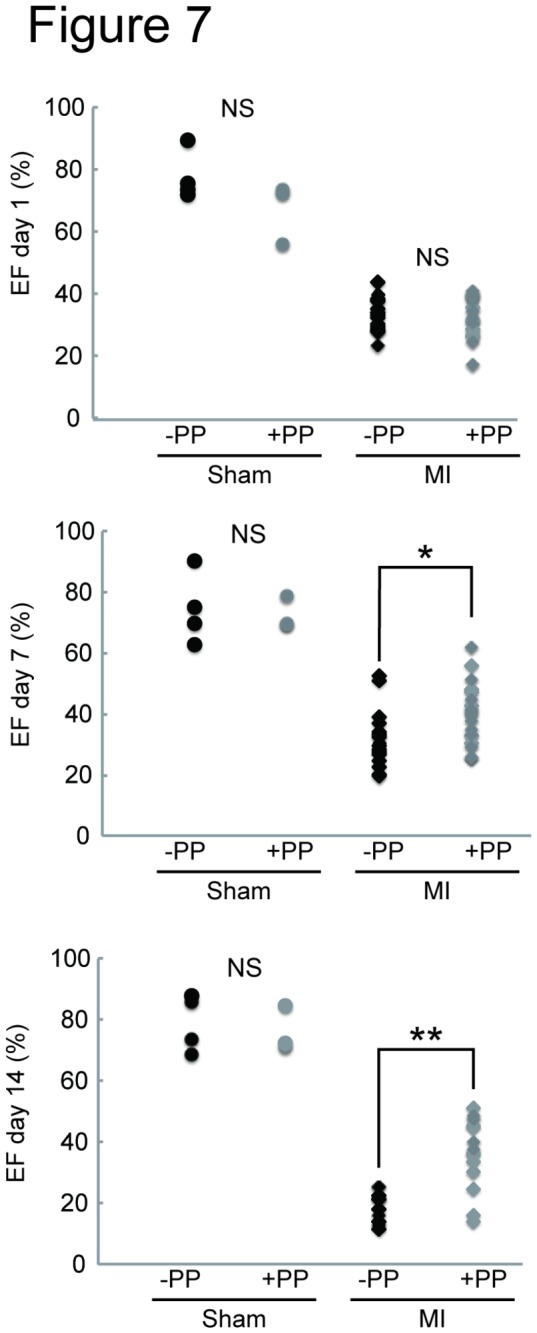
Improved cardiac contractility in the PP-treated infarcted heart in a mouse model of myocardial infarction. An effect of PP on cardiac function following myocardial infarction. Cardiac function following myocardial infarction was measured by echocardiography and shown as ejection-fraction (EF: %). The mice treated with vehicle (-PP) or PP (+PP) were subjected to echocardiography at days 1, 7 and 14 post sham-operation (Sham) or ligation (MI). The number of mice studied are as follows: Day 1: (Sham-PP: n=4; Sham+PP: n=3; MI-PP: n=17; MI+PP: n=20) Day 7: (Sham-PP: n=4; Sham+PP: n=3; MI-PP: n=17; MI+PP: n=20), Day 14: (Sham-PP: n=4; Sham+PP: n=3; MI-PP: n=11; MI+PP: n=14). ^★^p<0.05, ^★★^p<0.01. NS: not significant.

**Figure 8:**
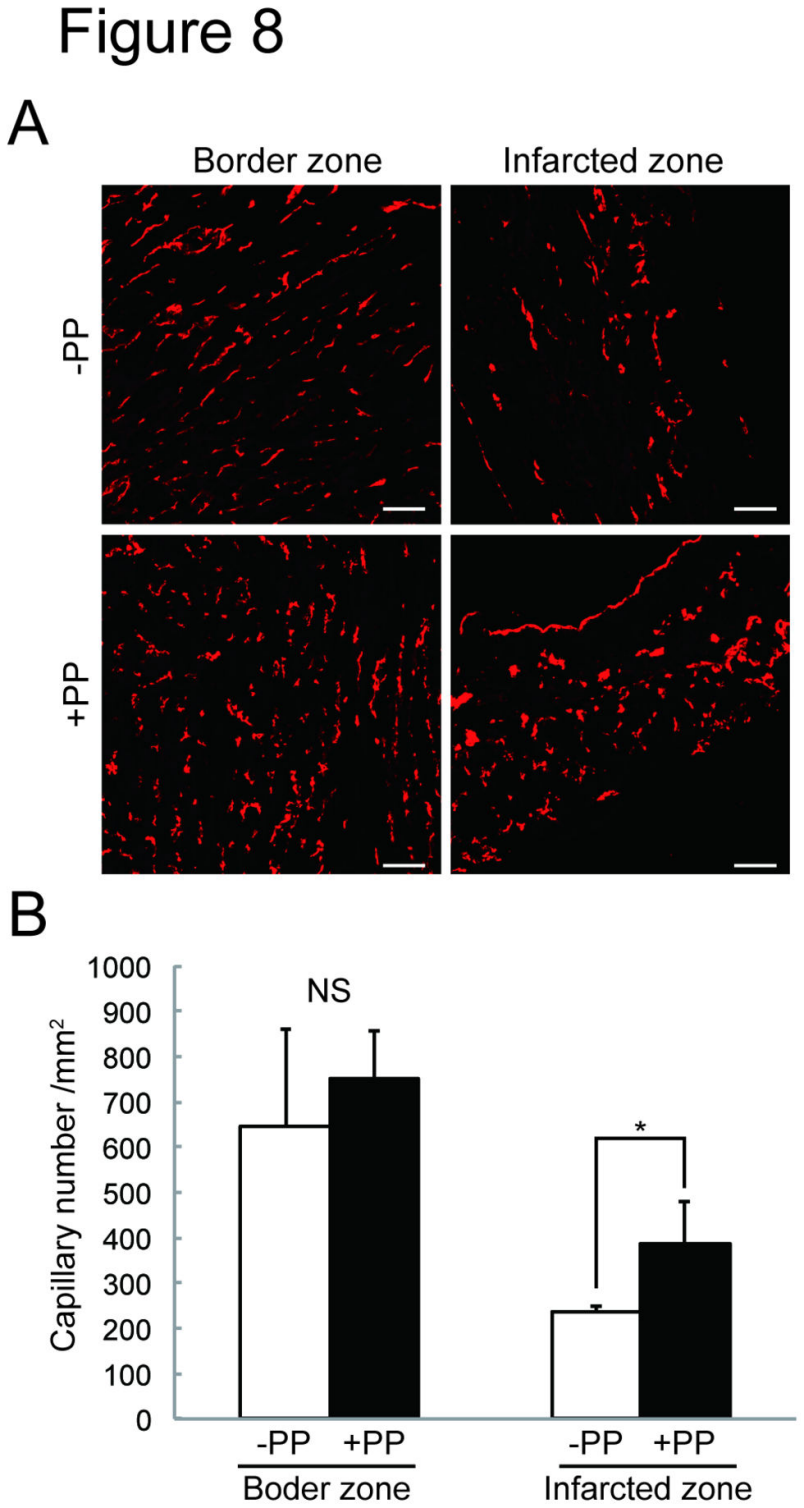
Increased microvascular density in the PP-treated infarcted heart in a mouse model of myocardial infarction. **A**. CD31^+^ capillaries in both border and infarcted zones in the scarred heart. The capillaries were identified with anti-CD31 antibodies in the cardiac sections prepared from the mice administered with vehicle (DMSO) (-PP) or PP (+PP). Scale bars: 50 μm. **B**. Quantitation of CD31^+^ capillary density. The data were collected from mice administered with vehicle (DMSO) (-PP) (n=3 mice) and with PP (+PP) (n=4 mice). CD31^+^ capillary density was measured as represented as capillary number/mm^2^. Data: average±s.d. ^★^p<0.05. NS: not significant.

We next tested the effectiveness of PP on cardiac fibroblasts *in vivo* by examining whether PP could thwart fibrosis by attenuating fibroblast survival in a mouse model of myocardial infarction (Figures 2 - 8). In this model, left coronary artery was permanently ligated by surgical ligation. Within 24 hr following the occlusion, most of the cardiomyocytes in left ventricle die due to ischemia, and cardiac fibroblasts begin to proliferate and migrate at 3 - 4 days-post-ligation, and the number of cardiac fibroblasts peaks at 7 days-post-ligation (Figure 2). Secretion and deposition of collagen fibers by these activated cardiac fibroblasts cause fibrosis (Figure 2). The oral administration of PP was initiated at 1 day-post-ligation (i.e. after most of the cardiomyocytes die due to ischemia) and continued daily (Figure 2). At 4 days-post-ligation, the number of proliferating cells in the ischemic region (i.e. infarcted zone) was quantified by staining a nuclear marker, Ki67, associated with cell proliferation using anti-Ki67 antibodies (Figure 3A, B). The result shows significant reduction of Ki67^+^ cells in the infarcted zones of the heart treated with PP (Figure 3A, B). This result indicates that PP suppresses cell proliferation in the ischemic region at the beginning of fibrosis phase. The number of cardiac myofibroblasts that actively participates in fibrosis was identified and quantified by staining them with anti-α-smooth muscle actin (αSMA) antibodies in both border and infarcted zones (Figure 4A, B). The result demonstrated a significant reduction in the αSMA^+^ cardiac myofibroblasts at 7 days-post-ligation in both border and infarcted zones where active cardiac fibrosis were present (Figure 4A, B). By 14 days-post-ligation, we found the reduced extent of fibrosis in the heart treated with PP (Figure 5A, B). This reduced fibrosis was not due to the reduced infarct area (due to reduced cardiomyocyte death/necrosis), as no differences in the infarct size were found between the hearts treated with vehicle or PP at 4-days-post-ligation (Figure 5A, B). The reduced fibrosis in the infarcted heart treated with PP was further confirmed by the decreased level of hydroxyproline (quantitative indicator of mature collagen molecules) content in this heart (Figure 6). These results demonstrate that PP reduces the number of proliferating cardiac fibroblasts in the ischemic heart tissue *in vivo*, which results in the reduced fibrosis. Furthermore, by 14 days-post-ligation, the infarcted heart treated with PP exhibited improved left ventricular function as indicated by increased ejection fraction, as compared to that treated with vehicle only (Figure 7). These results show that the PP-treatment, not only reduces fibrosis, but also ameliorates myocardial contractile dysfunction, instead of causing cardiac rupture. 

One possible explanation for this beneficial (i.e., improved cardiac function), but not adverse (i.e., cardiac rupture), effect of the PP-induced reduction of fibrosis is that the decreased scar formation provided a permissive tissue environment for more efficient angiogenesis that is a favorable condition for contractile function of the remaining cardiomyocytes. In support of this possibility, we found a statistically significant increase in vascular density in the scarred heart treated with PP (Figure 8A, B). 

It was previously shown that PP had very little effect on cardiac remodeling, and was unable to reduce cardiac fibrosis or improve cardiac function [14]. The discrepancy between their result and ours may be due to the different protocols of PP administration to the mice. They directly injected PP into the heart at the same time when the coronary artery was ligated, thus several days prior to the proliferation of cardiac fibroblast and the initiation of fibrosis [14]. In our protocol, PP was orally administered beginning at 1-day-post-ligation, the time when most of the cardiomyocytes had already died, and the PP-administration was continued daily throughout the period of cardiac fibroblasts proliferation and fibrosis (Figure 2). It is possible that the beginning the PP-administration at one or two days later after the cardiomyocytes are dead, combined with the continuous daily administration therefore, may be the key to bring the beneficial effect of PP on thwarting fibrosis and improving cardiac function after myocardial infarction. It is important to note that the infarcted heart be treated by PP one or two days later from the time when the coronary artery was ligated to minimize any adverse effects on cardiomyocytes in ischemic region. We did not unnecessarily want to enhance the death of cardiomyocytes by PP by administrating this drug while the cardiomyocytes were still dying. For this reason, we began the PP-administration on the next day after the ligation (i.e., 1-day-post-ligation) (Figure 2).

In a previous study, it has been shown that the oral administration of PP with the same dosage as ours resulted in the clearance of the drug from the circulation within 12 hours [7]. Therefore, it would be of interest to quantitatively measure precise pharmacodynamics of PP in the infarcted mice, as such future study may allow pinpoint exactly the mechanism by which PP attenuate cardiac fibrosis following myocardial infarction in mice.

PP inhibits NADH-FR activities, and four non-NADH-FR pathways: canonical Wnt, UPR, androgen receptor and autophagy signaling pathways [6-10,15]. Which of these signaling pathways play the central role(s) in thwarting fibrosis in vivo remains for the future studies. In our study, we demonstrate that the inhibition of cardiac fibroblast proliferation by PP is dependent on glucose, and glutamine with lesser degree, but not oxygen concentrations (Figure 1B, C, D). Hence, it is less likely that the inhibitory effect of PP on cardiac fibrosis is mediated by the blocking of NADH-FR activity, as the activity is a part of the anaerobic respiratory chain in mitochondria. Androgen receptor knockout mice were previously shown to exhibit increased fibrosis [16], suggesting that the inhibition of androgen receptor signaling could not account for the effect of PP in a mouse model of myocardial infarction shown in this paper.

While cancer cells are widely known as “glucose-addicted”, they are also relatively resistant to glucose-starvation, the characteristics that they take advantage of for tumorigenesis [17-20]. Cardiac fibroblasts are also capable of surviving and proliferating under glucose-deficient condition and they exploit this unique ability to form fibrosis that impedes cardiac regeneration and interferes with normal cardiac contractility. Cancer cells also take advantage of their ability to survive under glucose-deficient condition for tumorigenesis [17-20]. These similar behaviors of cancer cells and cardiac fibroblasts may suggest that they share a related mechanism underlying their metabolic adaptation to nutrient stress. In this context, it is intriguing that PP also effectively kill several types of glucose-starved cancer cells, but its mode of action has been controversial [6,9,11-13]. Therefore, it is of interest to investigate a putative mechanism that might be shared between cardiac and cancer cells for their survival under glucose-starvation, which is targeted and inhibited by PP. 

In this paper, we demonstrate that PP compromise the survival of cardiac fibroblasts under glucose-deficient condition *in vitro* (Figure 1), and also attenuates fibrosis and ameliorates cardiac contractility without causing cardiac rupture in a mouse model of myocardial infarction (Figures 3 - 8). As PP is an FDA-approved anthelmintic drug, its therapeutic applications are likely to pose fewer problems. Hence, PP or other related drugs may serve as a novel therapeutics to thwart fibrosis and to facilitate the repair of scarred heart and possibly other scarred organs/tissues.

## Materials and Methods

### Isolation of Cardiac Fibroblasts

Cardiac fibroblasts were prepared as previously described [21]. Heart tissues were minced, washed by cold D-PBS (Ca^2+^, Mg^2+^ free), and suspended in 5 ml of 1 % collagenase (Worthington CLS-2) /DPBS (with Ca^2+^, Mg^2+^). Collagenase treatment was performed at 37 °C for 30 min. Each collagenase-digested sample was treated with 4 ml of 0.25 % trypsin (Invitrogen) at 37 °C for 5 min. Collagenase/trypsin-treated samples were further dissociated by pipetting up and down 50 times. Each tissue suspension was transferred to a 50 ml tube containing 20 ml of DMEM (Nacalai) supplemented with 10 % FBS, 1 % penicillin, 1 % streptomycin. Dissociated cells were centrifuged at 100 x g for 5 min at 4 °C and were suspended into DMEM supplemented with 20 % FBS, 1 % penicillin, 1 % streptomycin and cultured in the same media for approximately one week before being stored. 

### Cell Culture Experiments

Cardiac fibroblasts were plated on Collagen-Coated Dish (IWAKI). After 24 hr culture in the culture media (high glucose DMEM, 10 % FBS, 1 % penicillin, 1 % streptomycin), the cells were cultured in corresponding oxygen and nutrients conditions in the presence or absence of pyrvinium pamoate (6-(dimethylamino)-2-[2-(2,5-dimethyl-1-phenyl-1*H*-pyrrol-3-yl) ethenyl]-1-methyl-quinolinium pamoate salt, Sigma). Low glucose and glutamine conditions were accomplished by using DMEM without glucose and glutamine. As the culture media contained 10 % FBS, minute amounts of glucose and glutamine were present, of which final concentration was determined as 300 μM glucose, <100 μM glutamine. Low oxygen condition was accomplished by using Hypoxia chamber (Veritas) and premixed gas chamber (3 % O_2_, 10 % CO_2_, 87 % N_2_). Viable cells were identified by exclusion of Trypan Blue dye (GIBCO) and counted. 

### Animals

All animal protocols were approved by the Animal Care and Use Committee of Nara Institute of Science and Technology (Permit Numbers: 1123, 1223 and 1312). All experiments were conducted with 8- to 12-weeks old male ICR mice (CLEA Japan, Inc., Tokyo, Japan). 

### Myocardial Infarction

Coronary artery ligation was performed on ICR male mice (8–12 weeks-old) produce myocardial infarction as previously described [21]. Mice were anaesthetized by inhalation of 2 to 2.5% vaporized isoflurane (Abbott Japan) and intubated with a 20-gauge intravenous catheter. Animals were ventilated with a volume-controlled respirator (Harvard Apparatus) with 200 μl per cycle at a respiratory rate of 110 cycles min^-1^. After thoracotomy, the left coronary artery was ligated with an 8-0 nylon suture 1–2 mm below the tip of the left auricle. Occlusion was confirmed by the change of color (pallor) of the anterior wall of the left ventricle. The chest cavity and skin were closed with 5-0 silk sutures. The animals were extubated and allowed to recover from surgery on a warm plate for 1 hr. Sham-operated mice underwent the same procedure without tying the suture, but passing it below the coronary artery. Pyrvinium pamoate (PP) was suspended to a final concentration of 400 μg/ml in 4 % DMSO in saline, and the mice were force-fed intragastrically with 0.5 ml of the suspension or vehicle (4 % DMSO in saline). The oral administration was initiated at 1 day-post-ligation and continued daily up to 14 days-post-ligation. 

### Echocardiography

Echocardiography was performed on infarcted and sham-operated mice on days 1, 7 and 14 using Toshiba Diagnostic Ultrasound System machine (Aplio MX SSA-780A). In each instance, mice were anaesthetized with 2 to 2.5 % isoflurane and parasternal short-axis 2D-mode and M-mode views were recorded. The short- axis view was used to measure anteroposterior internal diameter, anterior wall thickness and posterior wall thickness at end-diastole and end-systole at the mid-papillary level. Left ventricular systolic function was assessed by ejection fraction: %EF =[(EDV - ESV) / EDV] × 100. All measurements and evaluations were performed in a blind controlled study.

### Hydroxyproline Assay

Hydroxyproline content of heart tissues was measured as previously described [21].

### Histology and immunohistochemistry

Whole hearts were harvested and embedded in O.C.T compound (SAKURA). Frozen sections (5μm thickness) were prepared and processed for Masson’s trichromes staining as previously described [22]. Sizes of necrotic and fibrosis were quantified using Image J (NIH) and BZ-9000 BZⅡ software (Keyence Japan, Inc.), respectively. Frozen sections were stained with anti-Ki67 (1:100) (Abcam), anti-CD31 (1:50) (BD Pharmingen), and Cy3-conjugated anti-αSMA (1:400) (Sigma) antibodies. Ki67^+^ and CD31^+^ signals were detected with Alexa 488 and Alexa 647 conjugated secondary antibodies (Invitrogen), respectively. Cell nuclei were counterstained with DAPI using ProLong® Gold antifade reagent with DAPI (Invitrogen). Ki67^+^ cells and capillary density were calculated by using Image J (NIH). αSMA-positive cells and αSMA^+^ area were calculated by using BZ-9000 BZⅡ (Keyence Japan, Inc.).

### Statistical Analysis

Statistically analyses were performed with Excel using Student’s t-test. Significance levels were set at and indicated as ^★^p<0.05, ^★★^p<0.01, ^★★★^p<0.001.
